# Supplementary material for: Effect of aging and Varroa parasitism on the paracellular and transcellular permeability of the honeybee blood-brain barrier
Source: PLoS One. 2026 Mar 27;21(3):e0343142. doi: 10.1371/journal.pone.0343142 (PMC13028543; doi:10.1371/journal.pone.0343142)
Supplement: Attachment — Submitted filename: PONE-D-24-43745_Response to Reviewers.docx [file pone.0343142.s002.docx]

**PONE-D-24-43745**

**Response to Reviewers**

Dear Dr. Rueppell,

Thank you for the opportunity to submit a revised draft of the manuscript titled “Effect of aging and *Varroa* parasitism on the paracellular and transcellular permeability of the honeybee blood-brain barrier” for publication in PLOS ONE. We greatly appreciate the time and effort that you and the reviewers committed to reviewing and providing feedback on our manuscript. We have incorporated the suggestions from the reviewers and yourself, which we feel has added significant value to the work. The changes we’ve made are in track changes in the manuscript, and have addressed each piece of feedback directly below in red.

**Editor Comments:**

1. I would like to ask you to perform a statistical power analysis and interpret your results accordingly.

We thank the editor for recognizing the merit of our simple yet complete study. We agree that a statistical power analysis and an according interpretation will strengthen the manuscript. We describe our methods (lines 197-212), results (lines 217-240, 241-250), and an interpretation of our results in the context of the power analysis in the Discussion section (lines 346-365).

In this new section, we provide more background on the experimental limitations, namely, our unequal sample sizes, that contribute to the power of our statistical analyses. We explain implications of variation in sample sizes in terms of our statistical analyses. We also draw the readers' attention to results that may have to be interpreted with caution.

2. Finally, you need to go through your writing thoroughly to eliminate any easily avoided mistakes (e.g., Line 118 or L. 175).

We also thank the editor for pointing out these errors. We have reviewed the manuscript closely to correct these and other easily avoided errors.

**Reviewer #1 Comments:**

1. In this regard, the number of animals is quite variable, ranging from 8 to up 22 honeybees. While limitations in animal availability are understandable, as shown in Table 1, comparing groups of 22 animals to 8 could obscure small differences. The authors should address this issue in the discussion section.

We thank the reviewer for their appreciation for the challenges of working with free-living animals (in this case honeybees developing with different, natural infection rates), and that balanced sample sizes cannot always be achieved in such contexts. The reviewer makes a strong point about laying out these implications for the readers, and to do this we have added a paragraph on sample sizes, and resulting statistical power, in the Discussion section (lines 346-365). In this new section, we provide more background on the experimental limitations resulting in our unequal sample sizes, and we explain implications of variation in sample sizes in terms of our statistical analyses. We also draw the readers' attention to results that may have to be interpreted with caution.

2. Similarly, in Fig. 1 and Fig. 2, the data are presented as box plots. I strongly suggest that the authors include all points in the plots or provide supplementary tables showing all values. This is particularly relevant, as the authors used parametric statistics but displays median and interquartile ranges as whiskers. Additionally, bimodal distribution may be present in this type of data, making nonparametric statistics more appropriate for analysis.

We appreciate the suggestions the reviewer has provided regarding the presentation of our data. To the first point, we have overlaid all data points over the plots. All datasets had equal variances, and the only dataset that was not normally distributed was the data pertaining to the paracellular permeability of mite load groups (Fig 1B). We have applied the nonparametric Wilcoxon Rank-Sum test on this data. Finally, we present updated boxplots that display the mean.

3. Why was a 0-1 mites range used? Would it be more informative to include a completely mite-free (no-stress) control group for comparison?

This is a valid question, and one which we agree should be explained more clearly in the manuscript. The 0-1 mite group was used as a reference to represent minimal exposure to mites, with the understanding that bees emerging with 1 mite may have experienced some stress. This allowed us to assess the effects of higher mite loads (2-4 mites) relative to low-mite exposure conditions. However, we recognize this as a limitation in our ability to understand the effect of *any* mite exposure, and will include a 0 mite group in future studies. We have added this explanation in the Discussion section (lines 361-363).

4. Additionally, could the authors discuss the possibility of an inflammatory response due to parasite-induced stress? This may be particularly relevant, as studies on Drosophila indicate that inflammation can increase the BBB permeability.

We agree that this is both an interesting and important addition to our Discussion. We have expanded upon the implications of a potential mite-induced inflammatory response on BBB permeability in the Discussion at lines 434-342.

**Reviewer #2**

1. It remains unclear if and how many independent experiments have been performed. Have animals from different hives been probed? The number of animals probed is very small and indicates just one independent experiment, which is clearly not enough to draw any general conclusion.

We have provided further clarity in the Methods - Animals section that honeybees for these experiments were sourced from multiple distinct colonies across multiple seasons between Fall 2020 and Spring 2022 (line 117).

2. The authors claim that increased transcellular diffusion over the BBB could have an effect on animal health. However, they do not show if this is really the case. Do bees with increased BBB transcellular permeability show a decrease in fitness? Or at least: Do bees that have been infected by multiple mites show a reduction in health span or fitness?

We thank the reviewer for bringing attention to the fact that we could improve the clarity that this study does **not** assess animal health. While we do not believe that we made such a claim, we have included further disclaimer that this study only assesses BBB integrity in the context of stress (lines 370-373). We did not remove any discussion that speculates on how BBB integrity *may* impact animal health, as we feel this is valuable to inform and encourage future studies that may seek to draw such connections.

3. The authors show that infection with more than two mites affects transcellular BBB permeability. They probe freshly emerged animals of the age of 1 day. However, it remains unclear if this phenotype persists. The phenotype might be an adaptation to the effect of mite infection during development (reduced resources) and might wear of rapidly after the animal got rid of the mites. In this case, it would probably not impact the animal’s health span or fitness. Thus, I suggest to analyze BBB permeability of animals that have been infected with mites during development at later stages of their life.

We thank the reviewer for this suggestion, which we will take it into consideration for future studies assessing the effect of *Varroa* on BBB integrity.

4. The authors suggest that a follow up study should increase the number of animals tested. I would strongly suggest to do this in this study.

Although larger sample sizes are always preferable, we experienced multiple practical limitations to further expanding the sample sizes for this study. That said, we have added additional discussion of sample sizes and resulting statistical power in the Discussion section (lines 346-365). In this new section, we provide more background on the experimental limitations resulting in our unequal sample sizes, and we explain implications of variation in sample sizes in terms of our statistical analyses. We also draw the readers' attention to results that may have to be interpreted with caution.

5. The authors probe bees of different ages, the oldest age group being 2-3 weeks old, if I am not mistaken. Hoe old does a worker be become usually? Is 2-3 weeks “old” for a bee? I suggest to probe the entire lifespan of a worker to be able to draw conclusions on aging.

We thank the reviewer for this comment – honeybee worker lifespan is extremely plastic, dependent on a variety of intrinsic and extrinsic factors, and aging is task-dependent rather than chronologically dependent. Physiological aging can be reliably detected in honeybee workers ~14 days after their first foraging flight, irrespective of the worker’s chronological age. For this reasons, we distinguish between “Young Foragers” and “Old Foragers” at this 14-day mark, as described in the Methods. We have clarified these unique aspects of honeybee aging in the Sample Collection section at lines 123-126.

6. The text (introduction and discussion) describing the features of the BBB contains several mistakes. E.g. the authors state that the BBB is enriched in active transport systems for the exchange of various substances (line 86). There are active transport systems, but many substances are also transported via facilitative transporters (e.g. glucose). Line 158 states that there are tight junctions between the cells – in insects the diffusion barrier is provided by septate junctions. In line 270 the authors claim that there is redundancy between different junctional proteins. Vast research in Drosophila suggests that the junctions are formed by big protein complexed, that comprise many different proteins, which are all needed to keep the complex stable (at least the core proteins). If one of them is missing the complex falls apart. Thus, I would not talk about redundancies when it comes to BBB protein complexes. There are more other imprecisions that should be corrected.
7. Several sentences suggest causative connections between unrelated information. Please review the text for inconsistencies and wrong causative relationships. In general, the text does need some proof reading.

**To 6 & 7:** We thank the reviewer for pointing out these inaccuracies. We have corrected these inaccuracies and have carefully reviewed the manuscript for any further inaccuracies, wrong causative relationships, and general proofreading.
