# Supplementary material for: Effect of aging and Varroa parasitism on the paracellular and transcellular permeability of the honeybee blood-brain barrier
Source: PLoS One. 2026 Mar 27;21(3):e0343142. doi: 10.1371/journal.pone.0343142 (PMC13028543; doi:10.1371/journal.pone.0343142)
Supplement: Attachment — Submitted filename: PONE-D-24-43745_Response to Reviewers_3.docx [file pone.0343142.s003.docx]

**PONE-D-24-43745**

**Response to Reviewers**

Dear Dr. Rueppell,

Thank you for the opportunity to resubmit a revised draft of the manuscript titled “Effect of aging and *Varroa* parasitism on the paracellular and transcellular permeability of the honeybee blood-brain barrier” for publication in PLOS ONE. We greatly appreciate the time and effort that you have committed to reviewing and providing feedback on our manuscript. We have incorporated your additional suggestions. The changes we’ve made are in track changes in the manuscript, and have addressed each piece of feedback directly below in red.

**Editor Comments:**

1. Simple check of spelling and grammar throughout (e.g., in the abstract, the statement "we assess a key dimensions of blood-brain barrier function")

We thank the editor for noticing this discrepancy and have review the entire manuscript to ensure that all other spelling, grammar, missing word, and other minor clarifying edits are fixed (lines 33, 34, 44, 60, 92, 96, 103, 108, 139, 152, 160, 164, 171, 180, 187, 190, 192, 204, 206, 210, 211, 223, 226, 231, 235, 250, 252, 253, 260, 261, 266, 267, 273, 274, 281, 289, 290, 296, 297, 306, 325, 329, 336, 341, 352, 364, 400, 410, 414)

2. The issue about your way of testing age or ageing: It needs to be clear in the abstract and discussion that you had limited control over the chronological age of workers bees, and that you are comparing age ranges that experienced varying amounts of foraging (although of course your groups are different on average). Whether you really have included "old workers" is not certain in the absence of better demographic data on your study subjects.

We thank the editor for pointing out this necessary clarification. We have made edits to the abstract (lines 35-36, 39, 41-42) and added two sentences to the discussion (lines 290-293, 337-340) that highlight this specific limitation in our sampling strategy.

3. The significance levels in figure 2 seem to be switched, indicating 2A as significant and 2B as N.S.

We thank the editor for pointing this out, we have made the relevant correction to Figure 2 (line 271).
